# Panretinal photocoagulation after or prior to intravitreal conbercept injection for diabetic macular edema: a retrospective study

**DOI:** 10.1186/s12886-021-01920-8

**Published:** 2021-04-01

**Authors:** Wei Zhang, Guiyang Zhao, Weijie Fan, Taihong Zhao

**Affiliations:** Department of Ophthalmology, Nanjing First Hospital, Nanjing Medical University, 68 Changle Rd, Nanjing, 210006 China

**Keywords:** Diabetic macular edema, Conbercept, Panretinal photocoagulation, Diabetic retinopathy, Anti-VEGF, Visual acuity, Optical coherence tomography

## Abstract

**Background:**

Panretinal photocoagulation treatment (PRP) have been known as a standard treatment for proliferative diabetic retinopathy (PDR) or severe nonproliferative diabetic retinopathy (sNPDR). However, there is no consensus on when PRP should be administrated if anti-VEGF treatment is needed for the concurrent diabetic macular edema (DME). This study is to evaluate the difference between two groups of PRP prior to, or after intravitreal conbercept (IVC) for patients with PDR or sNPDR combined with DME.

**Methods:**

This was a retrospective study. Fifty-eight eyes with DME secondary to PDR or sNPDR were divided into two groups; the PRP after (PRP-*after* group), or prior to (PRP-*prior* group), IVC. Changes in number of IVC injections, best corrected visual acuity (BCVA), and central subfield macular thickness (CSMT) were compared after 4 weeks, 12 weeks, 1 year, and 2 years from the first IVC injection.

**Results:**

The mean number of injections in PRP-*after* group was 4.8 (1 year) and 6.4 (2 year), lower than 6.4 (1 year) and 8.5 (2 year) in PRP-*prior* group (both *p* = 0.002). There was no significant difference in change in BCVA and CSMT between two groups after each follow-up.

**Conclusion:**

PRP after IVC requires less injections but also yields similar visual and anatomic outcome comparing with PRP prior to IVC in patients with diabetic retinopathy combined with DME.

**Supplementary Information:**

The online version contains supplementary material available at 10.1186/s12886-021-01920-8.

## Background

Proliferative diabetic retinopathy (PDR), the leading cause of vision loss for working populations, usually involves the breakdown of blood-retinal barrier and neovascularization, leading to increased permeability and leakage from retinal capillaries [1]. The leaked fluid, if accumulates within the retinal layers, resulting in a thickened macula, then called macular edema (ME). During the past three decades, panretinal photocoagulation treatment (PRP) has been standard for patients with PDR [2, 3], and the emerging adjunctive anti-VEGF agents have shown superior outcomes, especially for DEM secondary to PDR or NPDR [4–8].

In clinical practice, however, there is no consensus on when PRP should be administrated if anti-VEGF treatment is combined [9–11]. On one hand, PRP decreases metabolic demand in the peripheral retina and alleviates the ischemia that drives neovascularization [12], it is unknown whether or not standard PRP could reduce the number of anti-VEGF injection in the long run. On the other hand, PRP may aggravate macular edema due to retinal inflammation and increased vascular permeability [13], it is also unknown whether this PRP-induced macular damage is temporary or permanent.

Currently, five VEGF antagonists are available in clinic, as pegaptanib, bevacizumab, ranibizumab, aflibercept and conbercept. Conbercept (KH902; Chengdu Kanghong Biotech Co., Ltd., Sichuan, China) is a newly developed anti-VEGF drug and has been applied in clinic. Compared with bevacizumab and ranibizumab, conbercept can bind to all isoforms of VEGF-A, VEGF-B, and placental growth factor (PlGF). A number of studies have demonstrated its high affinity in the treatment of wet age-related macular degeneration (wet-AMD) [14], macular edema secondary to retinal vein occlusion [15], DME [16], and the preoperative administration in PDR [17].

Given the uncertainty of the timing of PRP and anti-VEGF drugs, the present study is to determine PRP should be performed prior to, or after, IVC in the treatment of DME.

### Patients and methods

This study is a nonrandomized retrospective comparative study which recruited patients with DME between June 2018 and April 2020. The study adhered to the tenets of the Declaration of Helsinki and was approved by the Institutional Review Board (IRB) at the Nanjing First Hospital, Nanjing Medical University. Since all data collected were retrospective, patient informed consent was not required by the IRB. Patient data were deidentified in order to protect patient privacy.

### Patient eligibility

Inclusion criteria included: (i) patients over 18 years of age; (ii) with DME secondary to severe non-proliferative diabetic retinopathy (NPDR) or PDR; and (iii) treated with the combined therapies. PDR is defined as diabetic retinopathy with neovascularization after fluorescence fundus angiography (FFA) [3]. Severe NPDR is diagnosed with one or more of the following: hemorrhage in four quadrants, venous beading in two quadrants, and/or intraretinal microvascular abnormalities (IRMAs) in one quadrant [18].

Exclusion criteria included: (i) patients with history of prior anti-VEGF, laser or vitrectomy in the study eye; (ii) significant media opacities; (iii) neovascular glaucoma; (iv) uncontrolled hypertension, renal failure, or known coagulation abnormalities or current use of anticoagulative medication other than aspirin; or (v) insufficient data for analysis.

### Study design

Patients were divided into two groups according to the treatment regime. PRP-*after* Group received two time-points PRP (at weeks 1 and 3) 1 week after IVC injection, while PRP-*prior* Group firstly received PRP at two time-points (at weeks 0 and 2) according to ETDRS guidelines [19] and IVC was then administrated the day after the completion of PRP session.

PRP was performed using an argon laser (532 nm) laser (LIGHTLas TruScan 532 Laser with built-in slit lamp biomicroscope; Quantel Medical, Cournon d’Auvergne Cedex, France) with the aid of an OMRA-PRP 165 (US Ophthalmic, Doral, FL, USA) ocular contact lens. The PRP technique was performed for a minimum of 1500 standard argon confluent laser (532 nm) burns (two sessions) with a spot size of 200 μm spot size, pulse duration of 20 ms, interval 100 ms, and power of 150–250 mJ, resulting in typical grey-white lesions. All patients received intravitreal injection of conbercept (0.5 mg/0.05 mL, Chengdu Kanghong Biotech, Inc., Chengdu, Sichuan, China) in the inferior-temporal sector 4 mm from the sclerocorneal limbus.

Each patient received at least 3 IVC injections as a loading phase and followed by pro re nata (PRN) injections with regular monthly monitoring for 24 months. Criteria for retreatment included (i) further reduction in BCVA due to DME persistence or progression, or (ii) central retinal thickness gain by ≥20% as compared to best value ever, or (iii) central retinal thickness > 250 μm.

At weeks 12 and 1 year, if active new vessels were detected on fluorescein angiography, patients received five hundred 500-lm additional spots per quadrant of active new vessels.

Patients were followed at baseline, 4 weeks, 12 weeks, 1 year, and 2 years after the first IVC injection.

### Data collection

At baseline and each follow-up, patients underwent a detailed ophthalmologic examination including measurement of the logarithm of the minimum angle of resolution (logMAR) Snellen best corrected visual acuity (BCVA), intraocular pressure (IOP), dilated slit-lamp biomicroscopic examinations, color fundus photography, FFA, and OCT.

The main outcome measured was the number of IVC injections. Secondary outcomes measured included logMAR BCVA and central subfield macular thickness (CSMT). Macular OCT was performed using a Heidelberg Spectralis Machine (Heidelberg Engineering GmbH, Heidelberg, Germany) to quantify CSMT. CSMT, also known as foveal thickness, was defined as the average thickness of the macula in the central 1 mm ETDRS grid [20].

### Statistical analysis

All analyses were performed using SPSS 20.0 (SPSS, Inc., Chicago, IL, USA). Chi-square test was used to compare baseline differences in categorical data. Continuous variables were firstly checked for normality using Kolmogorov-Smirnov test. Nonparametric data were expressed median and range and analyzed by Kruskal-Wallis variance analysis. Continuous parametric data are presented as means ± standard deviation of the mean and were compared using an independent t test. If a calculated *P* value was smaller than 0.05, the difference was with statistical significance.

## Results

A total of 58 eyes of patients with severe NPDR or PDR were included, 28 in PRP-*after* Group and 30 in PRP-*prior* Group. The patients’ age ranged from 38 to 74 years of age (mean, 54.8 ± 9.1) in PRP-*after* Group and 31–76 years of age (mean, 53.5 ± 10.3) in PRP-*prior* Group. There were 17 severe NPDR and 11 PDR in PRP-*after* Group, while14 severe NPDR and 16 PDR in PRP-*prior* Group (*p* = 0.284). Table 1 showed detailed patient demographics and baseline characteristics. There was no significant difference in baseline gender ratio, age, type of DM (Type 1/Type 2), duration of DM, HbA1c level, logMAR BCVA, and CSMT between the two groups.

**Table 1:** Baseline clinical characteristics

Characteristics	PRP-*after* group	PRP-*prior* group	*P* value
Number of eyes	28	30	
Gender (M/F)	12/16	19/11	0.118
Age of onset (years; mean ± SD)	54.8 ± 9.1	53.5 ± 10.3	0.618
Type of DM (Type 1/Type 2)	4/24	7/23	0.508
Duration of DM (years; mean ± SD)	13.0 ± 5.2	13.4 ± 5.3	0.756
HbA1c (mean ± SD)	7.4 ± 0.6	7.3 ± 0.5	0.510
Presence of hypertension	11/17	12/18	0.956
Treatment regimen (no insulin/insulin)	6/22	8/22	0.641
DR severity (Severe NPDR/PDR)	17/11	14/16	0.284
Preoperative BCVA (LogMAR)	0.88 ± 0.30	0.80 ± 0.27	0.286
Preoperative CSMT (μm)	358.75 ± 98.70	389.27 ± 137.25	0.338
EZ (Intact/Disrupted)	10/18	13/17	0.553

*PRP* Panretinal photocoagulation, *DM* Diabetic mellitus, *NPDR* Non-proliferative diabetic retinopathy, *PDR* Proliferative diabetic retinopathy, *BCVA* Best-corrected visual acuity, *logMAR* Logarithm of minimal angle of resolution, *CSMT* Central subfield macular thickness, *EZ* Ellipsoid zone

After 1 year, the mean number of injections in PRP-*after* group was 4.9 ± 0.95 [95% CI, 4.52–5.62], less than 6.4 ± 2.37 [95% CI, 5.54–7.31] in PRP-*prior* group (*p* = 0.002). After 2 years, the mean number of injections in PRP-*after* group was 6.4 ± 1.5 [95% CI, 5.85–7.01], less than 8.5 ± 3.2 [95% CI, 7.38–9.76] in PRP-*prior* group (*p* = 0.002). There were 8 patients in PRP-*after* Group (28.6%) and 9 in PRP-*prior* Group (33.3%, *p* = 0.61) receiving additional peripheral laser photocoagulation under the guidance of FFA.

**Fig. 1:**
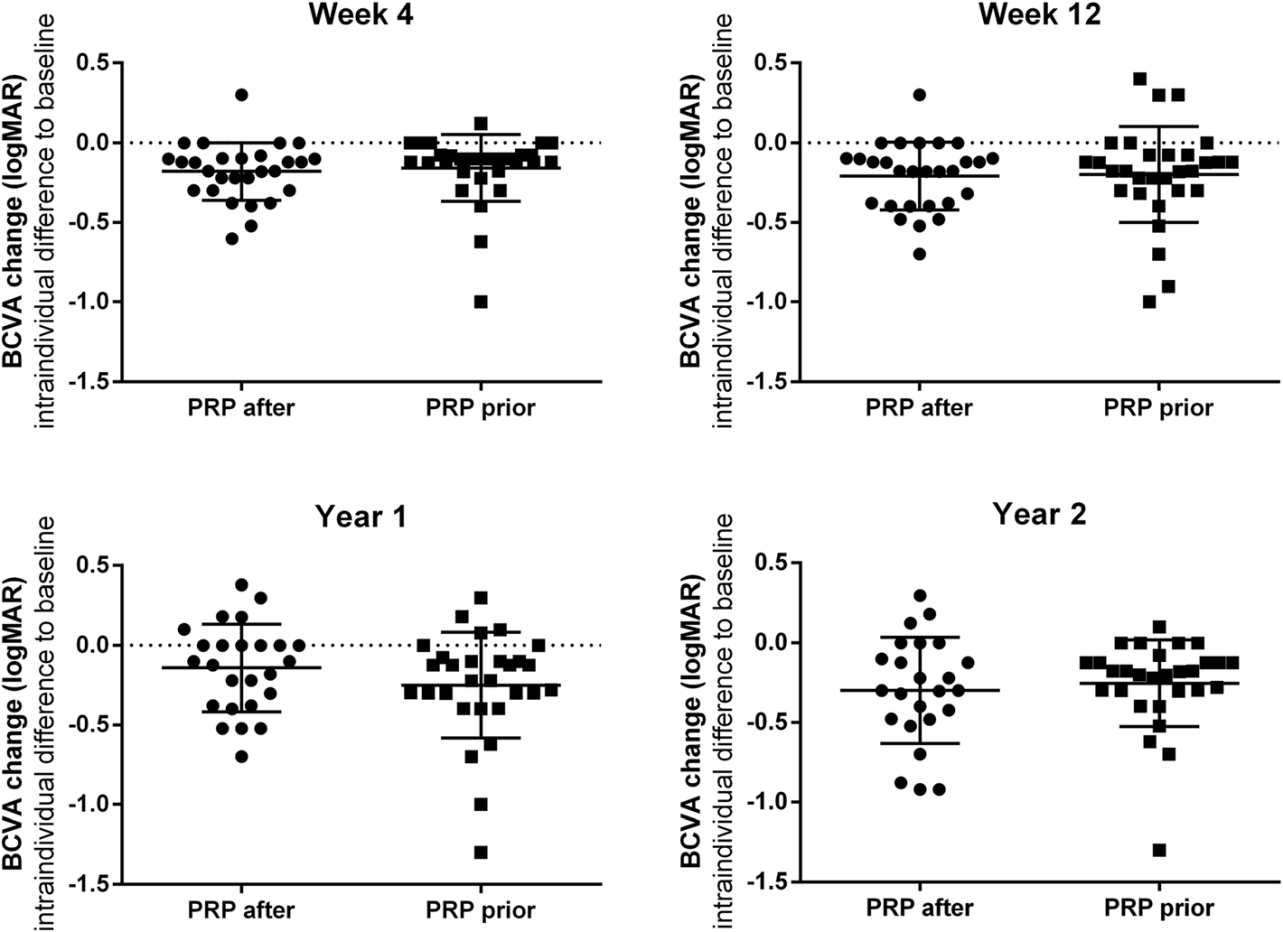
LogMAR BCVA improvement between PRP-*after* group and PRP-*prior* group. BCVA improvement was defined as the difference of logMAR BCVA between follow-up and baseline. There is no difference between the two groups at week 4 (*p* = 0.666), week 12 (*p* = 0.891), year 1 (*p* = 0.200), and year 2 (*p* = 0.602). BCVA: best corrected visual acuity; PRP: panretinal photocoagulation

In this long-term follow-up of 2 years, both groups gained significant VA improvement (*p* < 0.001, BCVA_follow-up_ vs BCVA_baseline_, supplementary file 1A). There was no significant difference in logMAR BCVA improvement between two groups at week 4 (*p* = 0.666), week 12 (*p* = 0.891), year 1 (*p* = 0.200), and year 2 (*p* = 0.602) (Fig. 1, Supplementary file 2A).

**Fig. 2:**
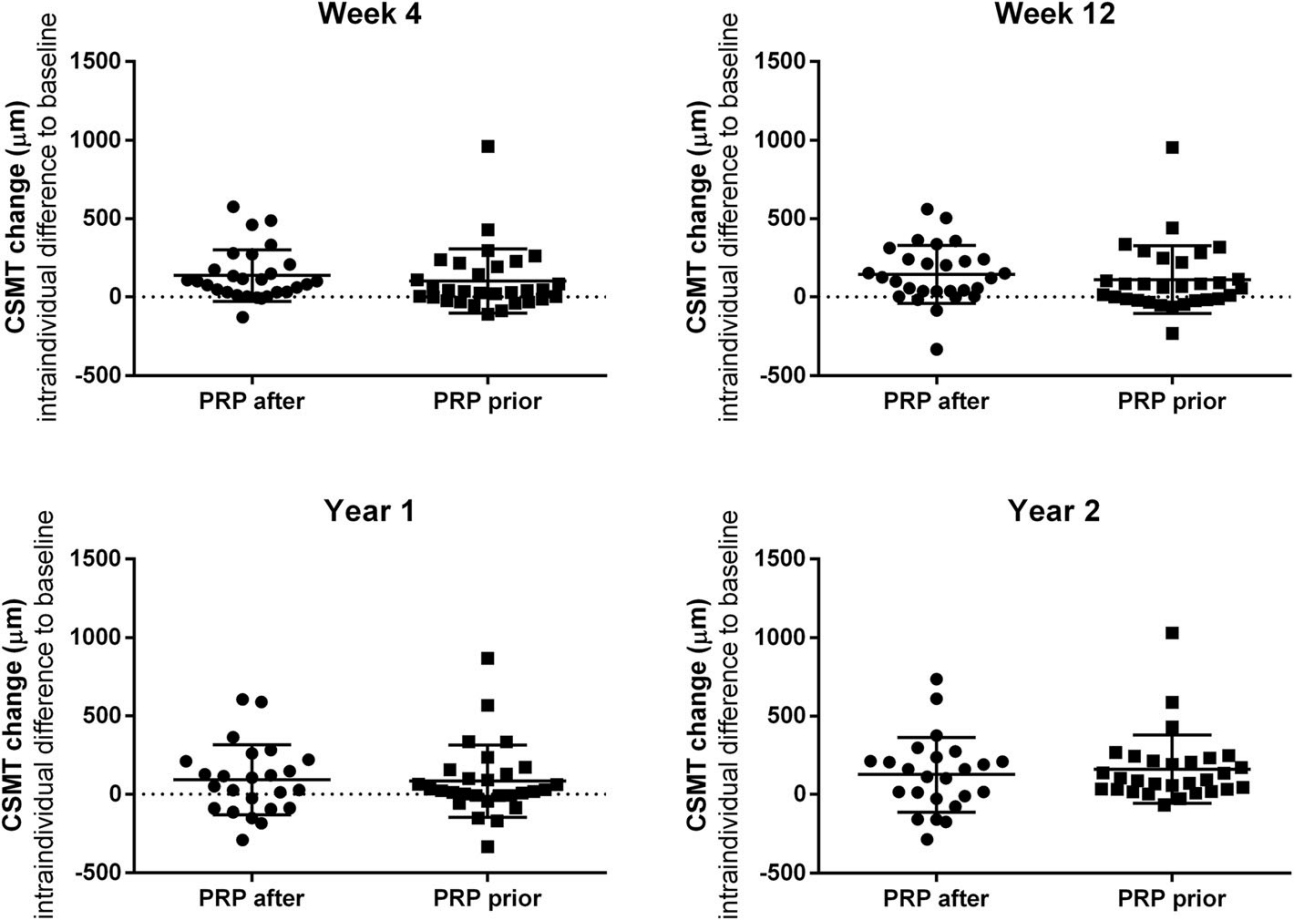
CSMT reduction between PRP-*after* group and PRP-*prior* group. There is no significant difference were detected in CSMT reduction between two groups at week 4 (*p* = 0.482), week 12 (*p* = 0.537), year 1 (*p* = 0.900), and year 2 (*p* = 0.586). CSMT: central subfield macular thickness; PRP: panretinal photocoagulation

The OCT shows the significant relief of macular edema on both groups after treatment (*p* < 0.001, BCVA_follow-up_ vs BCVA_baseline_, supplementary file 1B). However, no significant difference were detected in CSMT reduction between two groups at week 4 (*p* = 0.482), week 12 (*p* = 0.537), year 1 (*p* = 0.900), and year 2 (*p* = 0.586) (Fig. 2, Supplementary file 2B).

## Discussion

Results of the present study suggest that both treatment regime (PRP prior to, or after IVC) are associated with significant regression of DME and BCVA improvement in patients with DEM secondary to severe NPDR or PDR. However, the use of PRP after IVC was associated with a less IVC injections during a period of 2-year follow-up.

Patients with either severe non-proliferative DR (NPDR) or proliferative DR (PDR) can develop DME, which, if left untreated, causes vision impairment and legal blindness. For patients with PDR or sNPDR, PRP has been the standard of treatment for several decades [3, 21], while for DME, despite of the DR stage, intravitreal anti-VEGF therapy has emerged as first-line care for over 10 years [5, 16]. As known, photocoagulation leads to destruction of photoreceptors in the peripheral retina and decreases the demand of oxygen and metabolic of the retina, thus subsequently increasing the oxygen flow from the choroid to the inner retina [22]. However, PRP itself may also induce more inflammation and increase the severity of ME, temporarily or permanently decreasing vision quality [23]. As for anti-VEGF therapy, though evidence of Protocol S of the DRCR Retina Network has support the advantage of anti-VEGF therapy in the preservation of visual field 2 years after initiation of treatment [24, 25], their recent post hoc analysis also unexpectedly identified vision field losses in the ranibizumab group between years 2 and 5 [26]. The loss of vision field in ranibizumab group might be evidenced by diabetic retinal neurodegeneration progresses, the greater cumulative number of ranibizumab injections over time, and part of the natural history of PDR [26]. Considering that anti-VEGF treatment is expensive and requires high compliance with continuous, often monthly injections, PRP might still be the first-line treatment for PRP for years to come [24].

When DME is present, both anti-VEGF injections and PRP might be required to treat both DME and PDR. In clinic, however, there is no consensus on which one should be administrated prior to the other [9–11]. Anti-VEGF prior to PPR might favors the relief of macular edema, prompt recovery of visual acuity, and avoidance of DME severity induced by PRP. If anti-VEGF were administrated firstly, PRP cannot be planned within 1 week in consideration of infection possibility. In addition, IVC injection, as a minimally invasive surgery, requires family consent and internist examination to rule out the possibility of interfering the systemic conditions prior to treatment. Thus, IVC injection sometimes cannot be performed promptly after diagnosis. This study might be the first one to evaluate the difference between PRP prior to, or after IVC in the treatment of patients with PDR combined with DME. Interestingly, we found that IVC prior to PRP (PRP-after) needs less IVC after 1 and 2 years, yet the less IVC injections can also yield similar final functional and anatomic recovery. We speculate that the timely anti-VEGF injection can quickly inhibit retinal and vitreous VEGF activity, decrease vascular permeability, and facilitate the absorption of intraretinal or subretinal fluid. The accumulation of retinal fluid is responsible for the damage of photoreceptors, and this damage gets worse over time, which is associated with functional and anatomic outcome [27].

Recently, Arief et al [11] evaluated the difference in intravitreal bevacizumab (IVB) injection timing as adjuvant therapy to PRP in patients with DME secondary to severe NPDR and PDR, but detected no significant difference in changes in CSMT and BCVA between injection prior to and after PRP. In total, patients in our study gained an increase of logMAR BCVA from 0.83 ± 0.29 to 0.58 ± 0.25 (approximately 2 lines in Snellen chart, *p* < 0.001) after 2 years. Similarly, we also could not find the difference between two groups in CSMT and BCVA. Of note, in our study, we conducted a long-term follow-up and used number of injections as the primary outcome. The loading dose of anti-VEGF therapy in our study were 3 IVC injections while the study Arief et al [11] did not clarify this issue. After 1 year, the main number of IVC injections in PRP-*after* group was 4.9 ± 0.95, and 6.4 ± 2.37 in PRP-*prior* group (5.68 if combined both group), similar to previously report, which range from 4.5 to 6.74 [16, 28–30]. However, there has been no report concerning the IVC used in DME over 2-year follow-up (mean number of IVC were 7.53 combined all participants in the two groups).

The limited number of patients and retrospective property are major drawbacks of this study. A larger number of patients and a randomized controlled design are needed to elucidate the advantage of PRP after IVC regime. Besides, owing to the study design, patients in PRP-*prior* group actually got follow-up 3 weeks behind the PRP-*after* group. Lack of data, such as fasting blood sugar, HbA1c, and OCTA quantifications during the follow-ups is another concern. In addition, the different treatment regime actually led to different follow-ups in the two groups.

## Conclusions

The finding of this study underscores the order of anti-VEGF therapy and PRP in the treatment of naive PRP combined with DME. We found PRP after IVC requires less injections but also yields similar visual and anatomic outcome comparing with PRP prior to IVC in patients with diabetic retinopathy combined with DME. Further investigations are need to determine the changes in macular and peripheral vasculature, and intraocular (such as aqueous humor) VEGF level in patients undergoing the two treatment regimes.

## Supplementary Information

**Additional file 1: Supplementary file 1.** Mean BCVA and CSMT at each follow-up of the two groups. **Supplementary file 2.** Detailed data of BCVA and CSMT at each follow-up of the two groups**.**

## Data Availability

All data generated or analysed during this study are included in this published article.
