# Correction to: Panretinal photocoagulation after or prior to intravitreal conbercept injection for diabetic macular edema: a retrospective study

**DOI:** 10.1186/s12886-021-01971-x

**Published:** 2021-05-10

**Authors:** Wei Zhang, Guiyang Zhao, Weijie Fan, Taihong Zhao

**Affiliations:** Department of Ophthalmology, Nanjing First Hospital, Nanjing Medical University, 68 Changle Rd, Nanjing, 210,006 China

**Correction to: BMC Ophthalmology 21, 160 (2021)**

10.1186/s12886-021-01920-8

Following the publication of the original article [1], we were notified that the corresponding author was incorrectly marked. This should have been Dr. Zhang instead of Dr.Weijie.

The original article has been corrected.

## References

[1] Zhang, et al. Panretinal photocoagulation after or prior to intravitreal conbercept injection for diabetic macular edema: a retrospective study (2021). 2021;21:160. 10.1186/s12886-021-01920-8. 10.1186/s12886-021-01920-8 PMC8015169 33789617

