# Supplementary material for: Panretinal photocoagulation after or prior to intravitreal conbercept injection for diabetic macular edema: a retrospective study
Source: BMC Ophthalmol. 2021 Apr 1;21:160. doi: 10.1186/s12886-021-01920-8 (PMC8015169; doi:10.1186/s12886-021-01920-8)
Supplement: Supplementary file 1 — Additional file 1: Supplementary file 1. Mean BCVA and CSMT at each follow-up of the two groups. Supplementary file 2. Detailed data of BCVA and CSMT at each follow-up of the two groups. [file 12886_2021_1920_MOESM1_ESM.zip › supplmentary file 2.docx]

| **A. Mean BCVA at each follow-up of the two groups** | | | |
| --- | --- | --- | --- |
| BCVA (LogMAR, mean, 95%CI) | PRP-*after* group | PRP-*prior* group | P value |
| Baseline | 0.88 [0.77, 1.0] | 0.80 [0.70, 0.90] | 0.286 |
| Week 4 | 0.70 [0.57, 0.84] | 0.65 [0.55, 0.75] | 0.666 |
| Week 12 | 0.68 [0.54, 0.82] | 0.60 [0.49, 0.72] | 0.891 |
| Year 1 | 0.74 [0.60, 0.89] | 0.56 [0.45, 0.67] | 0.200 |
| Year 2 | 0.59 [0.45, 0.72] | 0.55 [0.45, 0.64] | 0.602 |

BCVA, best-corrected visual acuity; logMAR, logarithm of minimal angle of resolution; PRP, panretinal photocoagulation.

| **B. Mean CSMT at each follow-up of the two groups** | | | |
| --- | --- | --- | --- |
| CSMT (mean, 95%CI) | PRP-*after* group | PRP-*prior* group | P value |
| Baseline | 495.6 [436.0, 555.2] | 491.4 [415.7, 567.1] | 0.338 |
| Week 4 | 358.7 [320.5, 397.0] | 389.3 [338.0, 440.5] | 0.482 |
| Week 12 | 351.4 [300.5, 402.3] | 380.2 [328.4, 431.9] | 0.537 |
| Year 1 | 425.2 [357.0, 493.4] | 407.1 [343.5, 470.8] | 0.900 |
| Year 2 | 387.0 [312.6, 461.3] | 331.6 [298.6, 364.6] | 0.586 |

CSMT central subfield macular thickness; BCVA, best-corrected visual acuity; logMAR, logarithm of minimal angle of resolution; PRP, panretinal photocoagulation.
