# Supplementary figures and images for: Panretinal photocoagulation after or prior to intravitreal conbercept injection for diabetic macular edema: a retrospective study
Source: BMC Ophthalmol. 2021 Apr 1;21:160. doi: 10.1186/s12886-021-01920-8 (PMC8015169; doi:10.1186/s12886-021-01920-8)

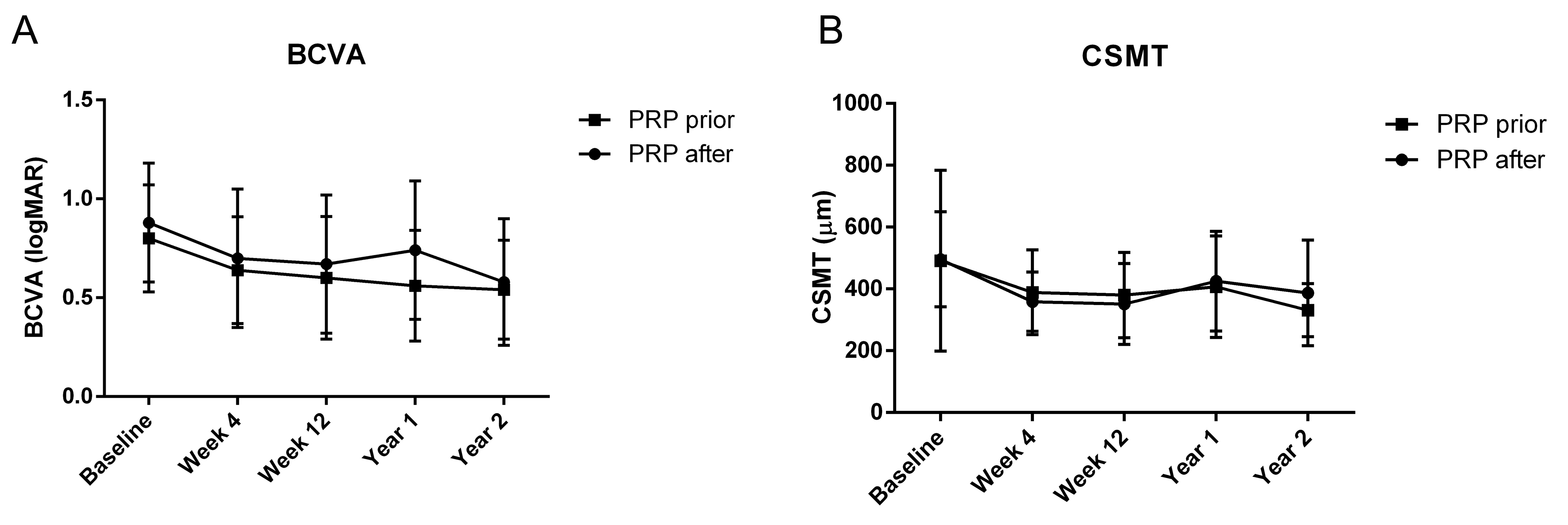

Supplement: Supplementary file 1 — Additional file 1: Supplementary file 1. Mean BCVA and CSMT at each follow-up of the two groups. Supplementary file 2. Detailed data of BCVA and CSMT at each follow-up of the two groups. [file 12886_2021_1920_MOESM1_ESM.zip › supplementary file 1.tif]
